# Identification of Targets from LRRK2 Rescue Phenotypes

**DOI:** 10.3390/cells10010076

**Published:** 2021-01-05

**Authors:** Joanne Toh, Ling Ling Chua, Patrick Ho, Edwin Sandanaraj, Carol Tang, Hongyan Wang, Eng King Tan

**Affiliations:** 1 Department of Research, National Neuroscience Institute, SGH Campus, Singapore 169856, Singapore; joannetoh85@gmail.com (J.T.); chua.ling.ling@sgh.com.sg (L.L.C.); patrick.ho.g.h@sgh.com.sg (P.H.); edwin_sandanaraj@sics.a-star.edu.sg (E.S.); 2 Singapore Institute for Clinical Sciences, Agency for Science, Technology and Research (A*STAR), Singapore 117609, Singapore; 3 Department of Research, National Neuroscience Institute, Singapore 308433, Singapore; carol_tang@nni.com.sg; 4 Duke NUS Medical School, Singapore 169857, Singapore; 5 Division of Medical Sciences, Humphrey Oei Institute of Cancer Research, National Cancer Centre, Singapore 169610, Singapore; 6 Neuroscience and Behavioral Disorders Program, Duke NUS Medical School, Singapore 169857, Singapore; hongyan.wang@duke-nus.edu.sg; 7 National University of Singapore Graduate School for Integrative Sciences and Engineering, National University of Singapore, Singapore 117456, Singapore; 8 Department of Physiology, Yong Loo Lin School of Medicine, National University of Singapore, Singapore 117597, Singapore; 9 Department of Neurology, National Neuroscience Institute Singapore, Singapore 308433, Singapore; 10 Department of Neurology, National Neuroscience Institute, SGH Campus, Singapore 169856, Singapore

**Keywords:** neurodegeneration, Parkinson’s disease, RNA sequencing, *Drosophila melanogaster*, LRRK2

## Abstract

Parkinson’s disease (PD) is an age-dependent neurodegenerative condition. Leucine-rich repeat kinase 2 (LRRK2) mutations are the most frequent cause of sporadic and autosomal dominant PD. The exact role of LRRK2 protective variants (R1398H, N551K) together with a pathogenic mutant (G2019S) in aging and neurodegeneration is unknown. We generated the following myc-tagged UAS-LRRK2 transgenic Drosophila: LRRK2 (WT), N551K, R1398H, G2019S single allele, and double-mutants (N551K/G2019S or R1398H/G2019S). The protective variants alone were able to suppress the phenotypic effects caused by the pathogenic LRRK2 mutation. Next, we conducted RNA-sequencing using mRNA isolated from dopaminergic neurons of these different groups of transgenic *Drosophila.* Using pathway enrichment analysis, we identified the top 10 modules (*p* < 0.05), with “LRRK2 in neurons in Parkinson’s disease” among the candidates. Further dissection of this pathway identified the most significantly modulated gene nodes such as eEF1A2, ACTB, eEF1A, and actin cytoskeleton reorganization. The induction of the pathway was successfully restored by the R1398H protective variant and R1398H-G2019S or N551K-G2019S rescue experiments. The oxidoreductase family of genes was also active in the pathogenic mutant and restored in protective and rescue variants. In summary, we provide in vivo evidence supporting the neuroprotective effects of LRRK2 variants. RNA sequencing of dopaminergic neurons identified upregulation of specific gene pathways in the *Drosophila* carrying the pathogenic variant, and this was restored in the rescue phenotypes. Using protective gene variants, our study identifies potential new targets and provides proof of principle of a new therapeutic approach that will further our understanding of aging and neurodegeneration in PD.

## 1. Introduction

Parkinson’s disease (PD) is a progressive age-dependent neurodegenerative disorder that is pathologically characterized by the loss of midbrain dopaminergic (DA) neurons in the substantia nigra and the accumulation of Lewy body aggregates [1,2,3]. PD is a multifactorial disease influenced by the complex interplay between genetic and environmental factors. Mutations in LRRK2 are the most frequent cause of autosomal dominant familial PD. Leucine-rich repeat kinase 2 (LRRK2) is a large multidomain protein that consists of armadillo repeats, ankryn-like repeats, and leucine-rich repeats at the N-terminal domain, a central catalytic core that contains 2 enzymatic domains, the first being the GTP-binding Ras of complex (ROC) domain with a carboxy-terminal of Roc (COR) domain, the second being the kinase domain [4,5], and a WD40 domain at the C-terminus. Mutations in LRRK2 are the most frequent cause of autosomal dominant familial PD. G2019S is one of the most common LRRK2 mutations, affecting 5–6% of familial PD [6,7], especially in the Western population. Other pathogenic mutations and risk variants identified include I2020T [8], R1628P, and G2385R, among others [9,10,11,12,13]. Patients with LRRK2 mutations display indistinguishable symptoms and pathologies to those with idiopathic PD, but the underlying mechanism linking LRRK2 mutations to pathology remains unclear [14]. LRRK2 protein has been associated with a diverse set of cellular functions, including mitochondrial function [15,16,17], cytoskeletal function [18,19,20], autophagy [21,22], and various signaling pathways [23,24]. A toxic gain of function has been postulated, but caution is needed when inferring data from studies on mutant LRRK2 because not all mutations are associated with increased kinase activity [25]. For instance, G2019S has been consistently linked to enhanced kinase activity in our own experiments [26,27] as well as in published reports [28,29]. The effects on kinase activity by other mutations/variants are less clear [8,30,31,32].

Genome-wide studies have mainly focused on pathogenic and risk LRRK2 variants, emphasizing the potential that the augmented kinase activity of the G2019S mutant form of LRRK2 may have novel targets and act in novel pathways [33,34]. The pathogenic mutations reported to date lie within the kinase enzymatic core of LRRK2, suggesting dysregulation of LRRK2 activity either through regulating its own autophosphorylation or other substrate phosphorylation at the serine/threonine site. Those mutations that lie in the ROC domain can impair GTPase activity and, in turn, affect kinase activity [35]. Multiple studies have focused on increased kinase activity in these mutants, with a view to identifying LRRK2 inhibitors as a therapeutic treatment. We and others have identified protective coding variants of LRRK2 (N551K and R1398H) that are associated with a decreased risk of PD [36,37,38,39]. However, the clinical association is correlative and is not sufficient to provide direct evidence for a cause-and-effect relationship. In vivo phenotypic characterization of these protective variants has been scarce. Only R1398H has been reported to affect GTPase function, axon outgrowth, and Wnt signaling, opposite to other LRRK2 pathogenic mutants [40,41], whereas N551K has not been reported. Previously, we have shown that a protective R1398H variant displayed diminished kinase activity compared to wild-type in the DA neuronal line [36]. Hence, we hypothesized that the protective coding variants of LRRK2 might suppress the effects of the LRRK2 risk variants or pathogenic mutations. If so, exploring the mechanism of action of these protective variants might help identify potential neuroprotective targets of LRRK2 in PD.

We generated a transgenic genetic model in *Drosophila melanogaster*, providing evidence that these protective variants confer a neuroprotective effect in the context of the pathogenic G2019S LRRK2 allele, a more common and widely studied variant. We identified transcripts differentially expressed in dopaminergic neurons under these conditions to identify potential targets and affected pathways through which this protective effect is mediated. Our RNA-sequencing data can provide a rich resource to identify neuroprotective targets against PD caused by LRRK2 mutations.

## 2. Materials and Methods

### 2.1. Fly Stocks

The following flies were used in this study: *tyrosine hydroxylase (TH)*-GAL4, *dopa decarboxylase* (*ddc*)-GAL4, *yellow white (yw)*, and UAS-UPRT flies (Bloomington *Drosophila* Stock Center. Flies were raised on standard yeast–cornmeal–agar medium at 25 °C with a 12-h light and dark cycle.

### 2.2. Generation of Transgenic Strains

Human LRRK2-expressing flies were created by generating transgenic human LRRK2 wild-type and variants, and point mutations were introduced into LRRK2 using Quikchange XL site-directed mutagenesis kitAgilent, Santa Clara, CA, USA#200516) and verified by sequencing to ensure the integrity of the cloned ORFs. PRDX2 plasmid (RC207413) was purchased from Origene. LRRK2 wild-type, N551K, R1398H, G2019S, N551K-G2019S, and R1398H-G2019S cDNA containing a myc tag at the C-terminus was inserted into the pUAST-attB plasmid, which allowed the UAS constructs to land into a chosen attP site in the fly genome during microinjection. Constructs were sent for microinjection into *Drosophila* embryos (BestGene Chino Hills, CA, USA).

### 2.3. Western Blot

Briefly, 40 to 50 heads were collected from flies and ground in M-PER mammalian protein extraction reagent buffer (ThermoFisher Scientific, Waltham, MA, USA, #78501, country) supplemented with protease inhibitor (Roche, Basel, Switzerland #11697498001) and PhosStop (Roche #4906845001) and placed on ice for 30 min. They were centrifuged at maximum speed for 15 min, and the supernatant was collected for Western blot. Protein was extracted from fly head homogenates, and equal amounts of protein from the various genotypes were resolved by SDS-PAGE and transferred onto nitrocellulose membrane using an Iblot 2 transfer device (ThermoFisher Scientific #IB21001 and #IB23002). The following antibodies are used for probing the blot for LRRK2: anti-GFP (Sigma Aldrich, St. Louis, MO, USA #G1544), anti-myc (Santa Cruz Biotechnology, Santa Cruz, CA, USA #sc-40)

### 2.4. Immunofluorescence and Confocal Microscopy

Flies were aged to Day 20 and Day 60 after eclosion before fly brains were dissected, fixed with paraformaldehyde, and stained according to published protocols [42]. Brains were probed with rabbit anti-tyrosine hydroxylase (1:500, Sigma-Aldrich #T2928). For the DA neuron count, the number of neurons in the five different clusters was scored under confocal microscopy and subjected to statistical analysis performed in Graph Pad Prism 6.

### 2.5. Climbing and Lifespan Assays

The drosophila climbing assays were analyzed using negative geotaxis assay on 20-, 40-, and 60-day-old flies. A cohort of 60 flies was separated into groups of 20 flies in individual 20-cm marked height-climbing columns. They were allowed to acclimatize in the climbing column for at least 30 min before the climbing test. The number of flies that managed to surpass the 20-cm mark in one minute was then recorded, and the tests were repeated thrice. To determine adult lifespan, 100 flies from each genotype, under the direction of *TH-GAL4* or *ddc-GAL4*, were maintained on standard media. Newly eclosed adult flies were transferred into vials containing fresh media every 3 days, and mortality was scored daily. Age-matched *TH-GAL4/*+ flies were used as controls.

### 2.6. TU Tagging

Female flies of indicated genotypes (wild-type, G2019S, N551K, R1398H, N551K-G2019S, and R1398H-G2019S were aged to 60 days in groups of 30, transferred to empty vials for 12 h, and then to 4TU-containing food for another 12 h. RNA was extracted using Trizol (Qiagen, Hilden, Germany #79306), and tagged RNA was purified as described [43]. One hundred fifty fly heads from each LRRK2 variant genotype (UAS-UPRT2.1-HA > THGAL4; UAS-LRRK2-6myc variants) were used for RNA sequencing experiment.

### 2.7. Library Preparation and RNA-Sequencing

The quality of samples was checked using an Agilent Bioanalyzer, and libraries were made with an Illumina TruSeq Stranded mRNA kit (#20020595). Prepped libraries were checked again using KAPA qPCR and an Agilent Bioanalyzer to ensure that the RIN value is at least 8 before being pooled and sequenced in the HiSeq High Output 1 × 76 bp.

### 2.8. Alignment Coverage Analysis

RNA-seq reads were obtained from 4 distinct fly phenotypes, including *LRRK2* wild-type (wt), *LRRK2* G2019S (pathogenic), *LRRK2* N551K and R1398H (protective), and N551K-G2019S and R1398H-G2019S (rescue) flies. Each experimental fly included 3 technical replicates. The processed fastq files were evaluated for quality metrics using FastQC (v0.11.1). Quality checking analysis ensured appropriate GC content without a hyper abundance of adaptors or duplicated sequences due to technical errors. The sequence reads profiled in the current study met standard quality metrics, as recommended by FastQC. Sample RNA-seq reads were aligned with *Drosophila melanogaster* reference genome version (BDGP release 5) using Top Hat v2.0.11 [44]. Bowtie2 was then employed to assemble transcripts by mapping and identifying splice junctions. For quality control, reads were only allowed to have up to 3 mismatches per 25 base pairs.

### 2.9. Differential Transcript Analysis

We used cufflinks (v2.2.0) to transform the aligned RNA-seq reads into predicted transcriptome assembly [45]. The mapped reads were evaluated for relative abundances, and fragments per kilobase of exon per million fragments mapped (FPKM) values were estimated for each fly phenotype. The statistical design for differential analysis is as follows: First, we used cuff merge, a functional routine in cufflinks to unify the predicted transcripts from the output of cufflinks evaluated in our statistical design. Subsequently, cuff diff function was invoked to determine the differential transcripts and isoforms across fly phenotypes. The cuff diff function identified the differential transcripts based on read counts and relative abundance in each transcript. The variance was estimated from technical replicates included in each fly system. A false discovery rate q-value of less than 0.05 was used to identify the differential transcripts across experimental comparisons (wild-type vs. mutant, mutant vs. protective, mutant vs. rescue experiments). We then used in-house R scripts to determine the transcripts that reflected true biological differences, that is, the transcripts that were altered pathogenically, which were reversed by our protective and rescue variants. We adapted logarithmic-fold change to capture the transcripts of interest. We performed visualization checks on transcriptome assemblies dispersion and differential abundance of isoforms using cummeRbund packages in R (v3.2) [46].

### 2.10. Pathway Analysis

We introduced an additional logarithmic fold change cutoff (>±0.3) to winnow down the differential genes for mapping to highly specific pathways. To identify key functional clusters, we used the DAVID functional annotation enrichment tool to aggregate key clusters mapped to functional annotation terms. The normalized enrichment score and enrichment *p*-values were extracted from the DAVID tool. The histograms were plotted for the mapped gene nodes in specific cluster terms to demonstrate the enrichment significance. The core gene nodes were interrogated with the Meta-Core pathway database to identify the genetic causal network connecting critical biological mechanisms.

### 2.11. Statistical Analysis

Quantitative data are expressed as mean ± SEM unless otherwise stated. Statistical significance for climbing assay and differences in the number of TH-positive DA neurons were analyzed using one-way ANOVA with Bonferroni’s post hoc test unless otherwise stated. The lifespan assay was analyzed with a log-rank test.

### 2.12. Real-Time PCR

For quantitative real-time RT-PCR in fly heads, total RNA was isolated with TriZol (Qiagen #79306), treated with DNase I (ThermoScientific #EN0521) for 30 min at 37 degrees to eliminate DNA contamination, and purified using the phenol/chloroform method. Equal amounts of cDNA were synthesized using random primers and MultiScribe™ Reverse Transcriptase (Applied Biosystems, Foster City, CA, USA #4311235). Real-time PCR with SYBR green detection was performed with GoTaq^®^ qPCR SYBR Green Master Mix (Promega, Madison, WI, USA, #A6001) using an Applied Biosystems^®^ 7500 Real-Time PCR System. The following primers were used: *Drosophila Jafrac1* (*PRDX2*) Fwd TCAACTCGTGCCGAAAGGTT, *Drosophila Jafrac1* (*PRDX2*) Rev TTTGCCCTTGTAGTCGCTCA, *RpS20* internal control Fwd TGTGGTGAGGGTTCCAAGAC, *RpS20* internal control Rev GACGATCTCAGAGGGCGAGT. No-template controls were included in each 96-well PCR reaction, and dissociation analysis was performed at the end of the run to ensure the specificity of the reaction. With fly heads, measurements were normalized to *RpS20*. The relative quantitation of Jafrac (*PRDX2)* expression levels was performed using the comparative Ct method.

## 3. Results

### 3.1. N551K and R1398H Variants Protected DA Integrity In Vivo

In vivo models of the N551K and R1398H protective LRRK2 alleles have not been reported. We generated transgenic *Drosophila* expressing the protective variants of *LRRK2* (N551K and R1398H) as well as flies expressing the protective alleles together with the pathogenic variant G2019S. Comparing the phenotypes of the flies carrying the pathogenic allele alone, the protective alleles alone or both together provide the experimental basis to address the hypothesis that the protective variants can suppress the phenotypic effects caused by the pathogenic LRRK2 allele.

**Figure 1:**
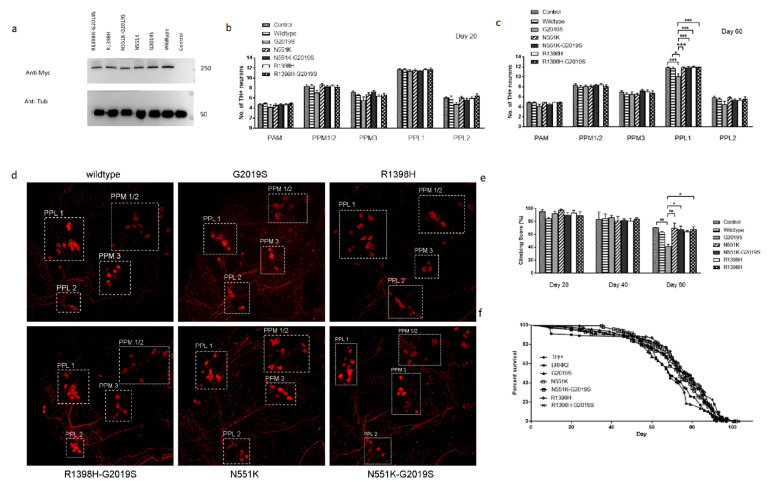
Expression of protective variants N551K and R1398H rescue pathogenic phenotypes caused by G2019S mutation. (**a**) Immunoblot of the various transgenes showing comparable LRRK2 expression in the various UAS-LRRK2 flies driven by the *TH-GAL4* driver. (**b**) Bar graphs show the number of TH-positive DA neurons in flies at 20 days after eclosion (*n* = 10, done in triplicates). (**c**) Bar graphs showing the number of TH+ neurons in flies 60 days posteclosion (*n* = 10, done in triplicates). (**d**) Representative magnified confocal images of whole-mount brains 60 days after eclosion. The different clusters of TH+ neurons are boxed up and labeled. (**e**) Bar graph shows age-dependent climbing scores of female flies at different days after eclosion. Percentage of flies that reached the top of the column after 1 min was counted (*n* = 20, done in triplicates). (**f**) Survival curves were plotted as a percentage of living flies (*n* = 100, done in triplicates). The statistics for median lifespan was performed with a log-rank (Mantex–Cox) test (*p*-value less than 0.05 is taken as significant).Significance indicated on the graph: * *p* < 0.05, ** *p* < 0.01, *** *p* < 0.001, **** *p* < 0.0001.

We generated the following myc-tagged *UAS-LRRK2* transgenic flies: wild-type LRRK2 as a control, as well as N551K, R1398H, G2019S mutant alleles and double-mutants carrying N551K with G2019S or R1398H with G2019S, and isogenized them. *TH-GAL4* flies were used to drive the expression of the various *UAS-LRRK2* transgenes in tyrosine hydroxylase positive (TH+) neurons. Immunoblot analysis using the myc-tag antibody was used to compare the expression level of the different transgenic forms of LRRK2 (Figure 1a).

The effect of expressing the different forms of *LRRK2* was characterized in vivo by comparing the survival of TH-expressing clusters of DA neurons at Days 20 and 60 of adult life (Figure 1b–d). At 20 days of age, there was no significant difference in the number of TH+ neurons in flies expressing any of the variants of LRRK2 in any of the 5 clusters examined. At 60 days, there was a significant loss of TH+ neurons in the PPL1 cluster in flies expressing the pathogenic variant G2019S compared to flies carrying the N551K, R1398H variants alone or together with the G2019S variant. This observation suggests that the presence of N551K or R1398H variants can counteract the pathogenic effect of the G2019S mutation when the two are present together. Together with studies from human PD patients [36,37,38,47], this finding supports the hypothesis that these alleles confer a protective function over risk variants.

As locomotor dysfunction is another indication for PD, we compared the mobility of the various genotypes. At 60 days posteclosion, we observed a marked reduction in the climbing abilities of G2019S flies compared to flies carrying the N551K or R1398H variants alone or together with G2019S (Figure 1e). This correlated with the protection of TH + DA neurons in rescue flies. In addition, we observed a significant decrease (*p*-value less than 0.05) in the median lifespan of G2019S flies when compared to the flies carrying the N551K or R1398H variants alone or together with G2019S (Figure 1f).

### 3.2. Molecular Analyses Using RNA-Seq Data Acquired from DA Neurons of Transgenic Fly Mutants Identify New Pathways and Targets

**Figure 2:**
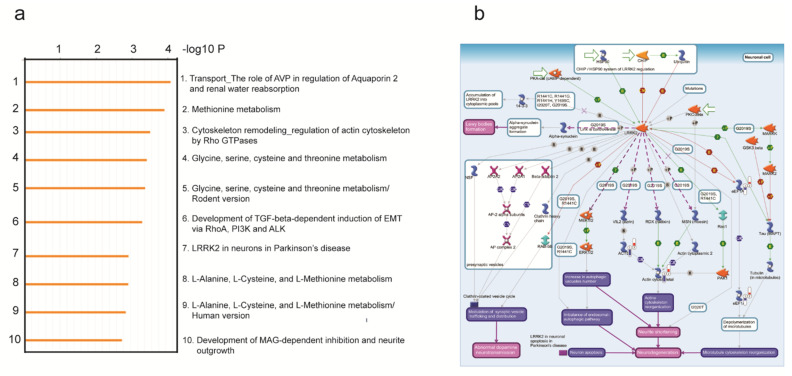
Metacore analysis on differential transcripts across *LRRK2* wild-type versus G2019S versus rescue flies. (**a**) List of top 10 significantly enriched pathways, including the *LRRK2* pathway (enrichment *p* = 0.001). (**b**) Metacore pathway map of *LRRK2* in neurons in Parkinson’s disease, including key gene nodes such as *eEF1A2*, *ACTB*, *eEF1A,* and actin cytoskeleton reorganization, which are significantly downregulated in the G2019S mutant and successfully restored in rescue flies from both N- and R-protective variants. Thermometer icons represent key gene nodes mapped from differential genes between wild-type versus G2019S and G2019S versus rescue flies. Blue color, downregulated; red color, upregulated.

**Figure 3:**
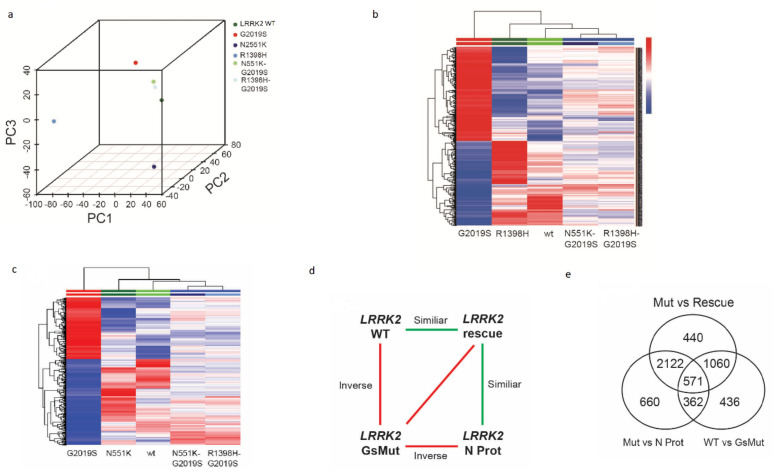
RNA-seq transcriptome analysis of *LRRK2* wild-type, pathogenic mutant and rescue variant flies (WT, G2019S, N551K protective and rescue flies by the N/R-protective variant). (**a**) Principal component analysis map of RNA-seq data acquired from fly variants: wild-type LRRK2, G2019S pathogenic variant, N551K and R1398H protective variants, N551K-G2019S and R1398H-G2019S rescue variants. (**b**) Heatmap of differentially regulated transcripts (*N* = 352) stratifying LRRK2 pathogenic variant from wild-type, R1398H protective, or R1398H-G2019S rescue variants. (**c**) Differential transcripts (*N* = 415) stratifying *LRRK2* pathogenic variant from wild-type, N551K protective, or N551K-G2019S rescue variants. (**d**) Statistical design evaluating RNA-seq experiments of *LRRK2* variant flies including N protective variant. Red color represents the model accounting for the differential transcripts having an inverse relationship. Green color denotes nonvariable transcripts between the respective biological fly phenotypes. (**e**) Venn diagram shows the number of differential transcripts across three statistical comparisons performed in the current study.

Previous studies have sought to dissect the molecular pathways responsible for mutant *LRRK2*-mediated PD utilized RNA sequencing (RNA-seq) data acquired from patient blood of idiopathic and LRRK2-G2019S carriers [48,49]. Several genes were identified to be functionally involved in processes known to be involved in PD pathogenesis, such as Akt signaling, glucose metabolism, or immunity, thus supporting the feasibility of such a molecular approach to explore key regulatory nodes. Similarly, we profiled our various fly mutants, which we earlier demonstrated to correlate with the functional activity associated with the development of PD-like symptoms. Specifically, we acquired RNA-seq data from TH neurons (via TU tagging method) [43] expressing *LRRK2* control and the R1398H or N551K, G2019S, and R1398H- or N551K-G2019S variants. We first determined the differentially regulated genes between the wild-type *LRRK2* and the G2019S pathogenic mutant. A pathway enrichment analysis identified the top 10 modules (*p* < 0.05), with “*LRRK2* in neurons in Parkinson’s disease” among the candidates (Figure 2a). Further dissection of this pathway identified the most significantly modulated gene nodes, such as *eEF1A2, ACTB, eEF1A,* and actin cytoskeleton reorganization (Figure 2b). In addition, our principal component analysis (PCA) map demonstrated that while the R1398H and N551K variants were distinct, the R1398H-G2019S and N551K-G2019S rescue variants clustered more closely with each other, as well as with wild-type *LRRK2* flies (Figure 3a), suggesting that both protective variants likely rescued the pathogenic phenotype through distinct mechanisms. Differentially regulated transcripts stratifying the LRRK2 pathogenic variant from wild-type, R1398H or N551K protective, or N551K-G2019S or R1398H-G2019S rescue variants are shown in the heat map (Figure 3b,c). Interestingly, closer examination of the differential transcriptomic patterns of the module “*LRRK2* in neurons in Parkinson’s disease” showed the inverse relationship between the pathogenic variant and R1398H, wild-type, R1398H-G2019S, or N551K-G2019S variants (Figure 3d). Amongst the differentially regulated modules were the GPCR family of proteins, including serotonin receptors, octopamine receptors, rhodopsin-like receptors, and the protein kinase (PKC) 1 pathway that was significantly active in the pathogenic mutant [19,50]. The induction of pathway genes was successfully restored by the R1398H protective variant and R1398H-G2019S or N551K-G2019S rescue experiments. The oxidoreductase family of genes was also active in the pathogenic mutant and resolved in R1398H protective and rescue variants. Retinoic acid biosynthesis pathway genes also demonstrated similar trends [51]. Collectively, the ability to enrich for established PD-related pathways between the wild-type, R1398H protective, and R1398H or N551K rescue variants validates our bioinformatics approach.

**Figure 4:**
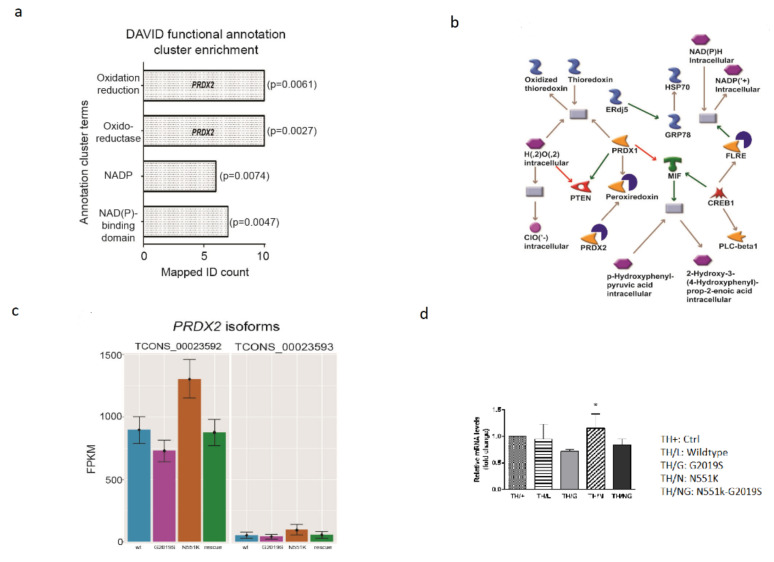
Validation of PRDX2. (**a**) Functional annotation enrichment on downregulated differential transcripts in pathogenic variant reveals significant enrichment of *PRDX2*-centred oxidoreductase pathway genes by the DAVID enrichment tool (nominal *p*-value <0.01). (**b**) MetaCore gene networks on the core nodes of oxidoreductase annotation clusters demonstrate *PRDX2* acts as a key player in *PTEN*, *CREB1,* and *FLRE* pathways. (**c**) Histogram plot shows differential FPKM levels across biological fly phenotypes for two *PRDX2* isoforms (TCONS_00023592 and TCONS_00023593; FDR *p* < 0.05). (**d**) Normalized Jafrac1 (Drosophila PRDX2 orthologue) mRNA levels measured by quantitative PCR in RNA isolated from 60-day-old fly heads. Data show mean and s.d. of three independent biological replicates. Significance on the graph: * *p* < 0.05.

As little is known about the function of the R1398H and N551K protective variants, our subsequent analyses focused on comparing transcriptomic differences between wild-type, G2019S, R1398H, and R1398H-G2019S, and N551K and N551K-G2019S variants. We identified 571 overlapping transcripts (Figure 3e), of which 415 genes satisfied the directional criteria described in Figure 3d. These differentially regulated transcripts demonstrated an inverse relationship between the G2019S pathogenic and wild-type or N551K protective or N551K-G2019S rescue or R1398H-G2019S rescue variants. We then performed functional annotation terms enrichment using the DAVID enrichment tool. Interestingly, we identified a significant enrichment of the oxidoreductase functional cluster comprising 11 gene nodes that were significantly modulated in mutants and successfully restored in rescue phenotypes (False Discovery rate (FDR) *p* < 0.05; Table 1). The altered oxidoreductase pathway included *DHDH*, *BLVRB*, *PRDX2*, *IDH3B*, *DBT*, *FAR1*, *AKR1D1*, *ME3*, *BBOX1*, *ACADSB*, and *MTHFD2L* genes. PRDX2 was among one of the most significantly altered genes identified between G2019S and N551K. We have recently shown that transgenic PRDX2 is able to rescue the LRRK2 pathogenic phenotype [27]. Intriguingly, PRDX2 has been previously shown to preserve cognitive function against age-linked hippocampal oxidative damage via signaling pathways involving CREB, CaMKII, and ERK. In support, we also did a further MetaCore analysis focused on human orthologs in literature-based evidence centered on the core nodes of oxidoreductase clusters (Figure 4a). It also revealed *PRDX2* as a key regulator of *PTEN*, *CREB1,* and *FLRE* pathways (Figure 4b). Finally, *PRDX2* sequences were recovered at a lower frequency in RNA from G2019S mutant flies compared to flies expressing wild-type *LRRK2* (Figure 4c; *p* < 0.05). *PRDX2* sequences were more abundant in N551K RNAs, as well as restored toward normal levels in flies expressing the double-mutant N551K + G2019S protein (rescue; *p* < 0.05).

We validated our RNA-seq analysis by examining Jaffrac1 a PRDX2 *Drosophila* orthologue mRNA levels through quantitative RT-PCR using RNA isolated from 60-day-old fly heads. We observed that Jaffrac1 (*PRDX2*) transcript levels decreased in RNA isolated from G2019S-expressing fly heads compared to those isolated from wild-type, N551K, and N551K-G2019S samples (Figure 4d), with a significant increase of Jaffrac1 (*PRDX2*) normalized expression levels in N551K compared to G2019S flies (*p <* 0.05).

## 4. Discussion

LRRK2 is a protein kinase commonly linked to autosomal-dominant familial PD. Although much has been reported on the pathogenic effects and mechanisms of LRRK2, there has been little research on the other variants of the protein that confer protective effects. Here, we provide evidence for the protective effects of the LRRK2 variants and explore their mechanism of action using a genetic in-vivo model. We showed that the R1398H and N551K alleles were able to suppress the pathogenic effects of the G2019S mutant of LRRK2 when both alterations were present in the same protein. New networks of genes and specific targets were identified through RNA-sequencing.

We identified a network of genes and specific targets from newly synthesized RNA in TH neurons that might have a neuroprotective effect on the G2019S mutation, with some of these networks substantiating previously reported in-vivo functional data on LRRK2. In particular, the MetaCore™ functional analysis of microarray, metabolic, SAGE, proteomics, siRNA, microRNA, and screening data revealed that the LRRK2 neuronal cell death pathway in PD is one of the top networks we have identified [52,53,54]. Others include the G-protein-coupled receptor (GPCR) family of proteins [50,55] and oxidoreductase [26,27,42] and retinoic acid biosynthesis modules [51,56]. Previous transcriptome studies on LRRK2 have revealed in various models that LRRK2 might be regulating proteins involved in the cell cycle, differentiation, the actin cytoskeleton, nervous system development, mRNA processing, ribosomal functions, long term potentiation, and calcium signaling pathways, among others [43,57,58,59]. The above studies are consistent with some of our identified genes. LRRK2 was previously shown to negatively regulate protein kinase A activity in *LRRK2*-enriched striatal projection neurons (SPN), supporting a pathogenic mechanism of SPN dysfunction in Parkinson’s disease (PD) [60]. LRRK2 also modulates retinoic-acid-induced neuronal differentiation of murine embryonic stem cells [51].

It is not clear how the protective variants (N551K and R1398H) confer neuroprotection. Aggregated N-terminal LRRK2 constructs have been shown to attenuate cell death induced by 6-hydroxydopamine (6-OHDA) [61]. However, the role of the N-terminal region of LRRK2 is still poorly studied, and it might have a role in protein–protein interaction in this region. Further studies to show how this mutation in the N-terminus might regulate kinase activity and affect interactions between different domains, dimerization of LRRK2, and toxicity will be of interest. LRRK2 can function as both GTPase and protein kinase, but the interplay between these two enzymatic domains is still not understood [62,63]. An R1398H mutation in LRRK2 can enhance GTPase activity, and this can lead to the impairment of kinase activity, providing evidence that the kinase activity of LRRK2 might be GTPase domain-dependent [64]. R1398H may lead to stronger Roc-COR dimerization and increased GTP hydrolysis, leading to a decrease in LRRK2 GTP-binding activity [40].

Among the list of differentially expressed genes that were restored in our rescue phenotype, we selected PRDX2 for further validation [27]. PRDX2 is a naturally occurring antioxidant, belonging to the family of redox enzymes that play an important role in health and disease. The PRDX family protects cells from oxidative-stress-induced apoptosis and has been associated with neurodegeneration [42,65]. Our current and published data provide corroborating evidence that the oxidoreductase family of genes is indeed among the genes that are being identified and restored. In conclusion, we provide in vivo evidence supporting the neuroprotective effects of LRRK2 variants (R1398H, N551K). The RNA sequencing of dopaminergic neurons derived from *Drosophila* expressing a pathogenic LRRK2 mutant (G2019S) identified upregulation of specific gene pathways that were restored in the rescue phenotypes. Using protective gene variants in rescue experiments, our study identified potential new targets and provided proof of principle of a new therapeutic approach for LRRK2-linked PD. Further functional validations of these targets can provide new insights into the mechanism of aging and neurodegeneration in PD.

## Figures and Tables

**Table 1:** List of functional clusters that were enriched using DAVID.

Category	Term	Count	%	*p*-Value	Ensembl ID	Gene Symbol	List Total	Fold Enrichment	FDR *p*-Value
SP_PIR_KEYWORDS	oxidoreductase	11	9.166666667	0.002426169	ENSG00000104808, ENSG00000090013, ENSG00000167815, ENSG00000101365, ENSG00000137992, ENSG00000197601, ENSG00000122787, ENSG00000151376, ENSG00000129151, ENSG00000196177, ENSG00000163738	DHDH, BLVRB, PRDX2, IDH3B, DBT, FAR1, AKR1D1, ME3, BBOX1, ACADSB, MTHFD2L	119	3.163745925	0.035624767
GOTERM_BP_FAT	GO:0055114~oxidation reduction	11	9.166666667	0.022170929	ENSG00000104808, ENSG00000090013, ENSG00000167815, ENSG00000101365, ENSG00000197601, ENSG00000122787, ENSG00000151376, ENSG00000129151, ENSG00000196177, ENSG00000163738, ENSG00000023909	DHDH, BLVRB, PRDX2, IDH3B, FAR1, AKR1D1, ME3, BBOX1, ACADSB, MTHFD2L, GCLM	103	2.260935625	0.467306665
INTERPRO	IPR016040:NAD(P)-binding domain	7	5.833333333	0.000634	ENSG00000104808, ENSG00000090013, ENSG00000101444, ENSG00000197601, ENSG00000151376, ENSG00000124217, ENSG00000163738	DHDH, BLVRB, AHCY, FAR1, ME3, MOCS3, MTHFD2L	117	6.644615385	0.169621933
SP_PIR_KEYWORDS	nadp	6	5	0.002759564	ENSG00000104808, ENSG00000090013, ENSG00000008130, ENSG00000197601, ENSG00000122787, ENSG00000151376	DHDH, BLVRB, NADK, FAR1, AKR1D1, ME3	119	6.216871364	0.037948511

## Data Availability

Data is contained within the article.
